# Dose optimization strategy of the tyrosine kinase inhibitors imatinib, dasatinib, and nilotinib for chronic myeloid leukemia: From clinical trials to real-life settings

**DOI:** 10.3389/fonc.2023.1146108

**Published:** 2023-04-05

**Authors:** Fang Cheng, Qiang Li, Zheng Cui, Mei Hong, Weiming Li, Yu Zhang

**Affiliations:** ^1^ Department of Pharmacy, Union Hospital, Tongji Medical College, Huazhong University of Science and Technology, Wuhan, China; ^2^ Hubei Province Clinical Research Center for Precision Medicine for Critical Illness, Wuhan, China; ^3^ Hubei Key Laboratory of Biological Targeted Therapy, Union Hospital, Tongji Medical College, Huazhong University of Science and Technology, Wuhan, China; ^4^ Department of Hematology, Union Hospital, Tongji Medical College, Huazhong University of Science and Technology, Wuhan, China

**Keywords:** tyrosine kinase inhibitors, dose optimization, chronic myeloid leukemia, dose reduction, treatment-free remission

## Abstract

With the advent of tyrosine kinase inhibitors (TKIs), the treatment prospects of chronic myeloid leukemia (CML) have changed markedly. This innovation can lengthen the long-term survival of patients suffering from CML. However, long-term exposure to TKIs is accompanied by various adverse events (AEs). The latter affect the quality of life and compliance of patients with CML, and may lead to serious disease progression (and even death). Recently, increasing numbers of patients with CML have begun to pursue a dose optimization strategy. Dose optimization may be considered at all stages of the entire treatment, which includes dose reduction and discontinuation of TKIs therapy. In general, reduction of the TKI dose is considered to be an important measure to reduce AEs and improve quality of life on the premise of maintaining molecular responses. Furthermore, discontinuation of TKIs therapy has been demonstrated to be feasible and safe for about half of patients with a stable optimal response and a longer duration of TKI treatment. This review focuses mainly on the latest research of dose optimization of imatinib, dasatinib, and nilotinib in CML clinical trials and real-life settings. We consider dose reduction in newly diagnosed patients, or in optimal response, or for improving AEs, either as a prelude to treatment-free remission (TFR) or as maintenance therapy in those patients unable to discontinue TKIs therapy. In addition, we also focus on discontinuation of TKIs therapy and second attempts to achieve TFR.

## Introduction

Chronic myeloid leukemia (CML) is a myeloproliferative tumor formed by clonal adult cases of leukemia (1). With the advent of tyrosine kinase inhibitors (TKIs) targeting BCR::ABL1, the therapeutic prospect of CML has changed markedly (2). The long-term survival of patients with CML in the chronic phase (CP) has become close to normal life expectancy (3). Imatinib, dasatinib and nilotinib are the most commonly used TKIs for CML patients in clinical practice in China.

Long-term treatment with TKIs is accompanied with various adverse events (AEs) that significantly affect the quality of life and compliance of patients with CML, and have the potential to cause significant disease progression and mortality. Severe AEs associated with second-generation TKIs have also been reported. These include pleural effusion (PE) and pulmonary hypertension induced by dasatinib (4, 5) as well as nilotinib-related dyslipidemia and arterial thrombosis (6, 7).

Recently, increasing numbers of patients with CML have begun to pursue a dose optimization strategy, which included dose reduction and discontinuation of TKIs therapy. Dose reduction of TKIs has been suggested to be safe and feasible, and to elicit an optimal response, in patients with CML. Also, the prevention and management of AEs must also be considered to improve patient compliance and reduce the risk of treatment interruption (8, 9). Fassoni and colleagues developed a patient data-based mathematical model which suggested that a reduction ≥50% of the full dose of a TKI did not exacerbate outcomes from long-term treatment (10). Importantly, the dose reduction of TKIs should be considered as early as possible, but the clinical benefit of this approach is controversial if chronic toxicity occurs, especially in some specific settings (11). Furthermore, some patients with a sustained deep molecular response (DMR, BCR::ABL1^IS^ ≤ 0.01%) can achieve relatively long-lasting safe discontinuation of TKIs therapy [i.e., treatment-free response (TFR)].

In recent years, several clinical trials and real-life practices have indicated that treatment discontinuation has become a new therapeutic goal for patients with CML who are stable and have a DMR (12, 13). However, about half of patients have molecular recurrence and need re-introduction of TKIs therapy. Imatinib was first applied to the treatment of CML two decades ago, and only 5%–10% of patients can maintain TFR (14). Eighty percent of patients continue to need long-term therapy with a TKI to achieve long-term survival (though 20% of them meet the conditions for treatment discontinuation) (15). Therefore, this review focuses mainly on the latest research on the dose optimization of the TKIs imatinib, dasatinib, and nilotinib in patients with CML. This information includes dose reduction (**Table 1**) and TFR (**Table 2**). In this way, we aim to provide important references for the formulation of individualized therapeutic regimen for patients with CML.

**Table 1:** Clinical trials evaluating different imatinib, dasatinib and nilotinib doses.

TKIs	Study	TKIs dose	Patients	Publication time
Imatinib	RIGHT trial (15)	800mg	115	2009
	Baccarani et.al (16)	800mg VS. 400mg	216	2009
	Cortes et.al (17)	800mg VS. 400mg	476	2010
	Michel et.al (18)	800mg VS. 400mg	422	2019
	Cervantes et.al (19)	300mg	246	2017
TDM-guide imatinib dose optimization	Lankheet et.al (20)	NR	109	2017
	Adeagbo et.al (21)	NR	126	2017
Dasatinib	DASISION (22)	100mg/day VS. <100mg/day	519	2017
	CA180-034 (23)	100mg qd *VS.* 140mg qd VS. 70mg bid *VS.* 50mg bid	670	2016
	Naqvi et.al (24)	50mg	81	2020
	DAVLEC (25)	20mg	52	2021
	Latagliata et.al (26)	100mg/day VS. <100mg/day	65	2016
	Iurlo et.al (27)	100mg/day VS. <100mg/day VS. >100mg/day	853	2018
TDM-guide imatinib dose optimization	Shin et.al (28)	NR	102	2021
	Rousselot et.al (29)	NR	287	2021
Nilotinib	ENESTnd (30)	300mg bid VS. 400mg bid	563	2016
	NILO-RED (31)	300/400mg bid VS. 300/400mg qd	67	2017
	ENESTswift (32)	300mg bid VS. 400mg bid	20	2018

TKIs, tyrosine kinase inhibitors; qd, once daily; bid, twice daily; NR, not report.

**Table 2:** Characteristics of TKIs discontinuation trials.

Study	N	TKI	Minimum TKIs duration(y)	Minimum DMR duration(y)	TFR rate	Resumed treatment
France in 2007 (33)	12	IM	1.875	UMRD≥2	50% in the first 5 months	positive BCR::ABL1 transcripts
STIM1 (34)	100	IM	3	UMRD≥2	43% at 6 months and 38% at 60 months	Significant increase of 1-log or loss of MMR
A-STIM (35)	80	IM	3	MR4≥2	64% at 24 months and 61% at 36 months	Loss of MMR, UMRD
STIM2 (36)	124	IM	3	DMR≥2	61.2% at 12 months	Loss of MMR
TWISTER (37)	40	IM	3	UMRD≥2	47.1% at 24 months	Loss of UMRD
KID (38)	90	IM	2	MR4.5>2	62.2% at 12 months and 58.5% at 24 months	Loss of MMR
ISAV (39)	108	IM	2	UMRD≥1	48% at 36 months	Loss of MMR
DOMEST (40)	99	IM	2	MR4≥2	70% at 6 months, 68% at 12 months, and 64% at 24 months	Loss of MR4
DADI (41)	63	DA	1	DMR≥2	49% at six months and 48% at 12 months	Loss of MR4
First-line DADI (42)	58	DA	3	DMR≥2	55% at 6 months	Loss of MR4
D-STOP (43)	54	DA	2	DMR≥2	62.9% at 1 year	Loss of MMR
DASFREE (44)	84	DA	2	MR4.5≥1	48% at 12 months and 46% at 24 months	Loss of MMR
ENESTfreedom (45)	190	NL	3	MR4.5≥2	51.6 at 48 weeks	Loss of MMR
STAT2 (46)	78	NL	2	MR4.5≥2	67.9% at 12 months and 62.8% at 24 months	Loss of MR4.5
ENESTop (47–50)	126	NL	3	MR4.5≥1	57.9% and 53.2% at 48 week and 96 week, 52.0% and 46% at 144 weeks and 192 weeks, 42.9% at 5 years	Loss of MMR or MR4
NILSt (51)	149	NL	NR	MR4.5≥2	The TFR rate was 60.9% at both 1 and 3 years	Loss of MR4.5
STOP-2G (52)	60	DA NL	3	MR4.5≥2	63.3% at 12 months and 53.7% at 48 months	Loss of MMR
LAST (53)	172	IM NL DA BO	3	MR4≥2	60.8% at 12 months	Loss of MMR
EURO-SKI (54)	755	IM NL DA	3	MR4≥1	61% at 6 months and 50% at 24 months	Loss of MMR
GIMEMA (55)	293	IM NL DA BO	7	DMR≥3	68% and 73% in imatinib and second-generation TKIs at 12 months and 62% at 34 months	Loss of MMR

TKI, tyrosine kinase inhibitor; IM, imatinib; DA, dasatinib; NL, nilotinib; BO, bosutinib; DMR, deep molecular response; MMR, major molecular response; TFR, treatment-free remission; UMRD, undetected minimum residual disease; MR, molecular response.

## Dose reduction

### Imatinib

Several clinical trials have explored the efficacy of high-dose imatinib (800 mg/day) treatment compared with standard-dose therapy (16–19). High-dose imatinib appeared to elicit a faster major molecular response (MMR, *BCR::ABL1* ^IS^ ≤ 0.1%), but the prevalence of MMR was similar at 1 years or 2 years between the two groups being assessed. However, an increased prevalence of severe AEs and worse compliance by patients was reported in the high-dose-imatinib arm. As a result, patients received imatinib at 800 mg/day initially which was later reduced to 400 mg/day. In addition, dose reduction was accompanied by a reduction in the prevalence of AEs and medical costs, and could improve patient compliance. Claudiani and coworkers (8) conducted a retrospective study of 246 patients with CML receiving treatment with a lower dose of a TKI (imatinib, n = 90; dasatinib, n = 88; nilotinib, n = 81; bosutinib, n = 39) after achievement of MMR because of intolerable AEs. A “lower dose” of a TKI (mg/day) was defined as 200 or 300 for imatinib, 70, 50, 40 or ≤20 for dasatinib; 400, 300, or ≤200 for nilotinib; 300, 200, or <200 for bosutinib. Their findings suggested that dose reduction should not be recommended as routine clinical practice, but could be an acceptable and safe option for patients who cannot tolerate a standard dose of a TKI. Cervantes and collaborators (56) found that a reduction to 300 mg/day in 43 patients with CML who received imatinib (400 mg/day) initially as first-line treatment with a sustained DMR improved tolerability significantly and maintained a DMR continuously.

Therapeutic drug monitoring (TDM) is gradually becoming a practical tool to achieve individualized medicine for patients receiving targeted drugs (57). Peng and colleagues showed that fixed-dose imatinib showed high inter-patient variability to plasma exposure in patients with CML (58). An imatinib concentration in plasma >1000 ng/mL in patients with CML can lead to a beneficial clinical outcome (59, 60). Therefore, a TDM-based dose-adjustment strategy could improve the efficacy, and reduce the toxicity and medical cost, of imatinib therapy (20). In daily practice, Lankheet and colleagues (21) monitored the proportion of patients who reached the target trough concentration (C_min_) of a TKI (imatinib, sunitinib, or pazopanib) after a TDM-based dose-adjustment strategy. The proportion of patients with the target C_min_ increased from 38% to 64%, which suggested that a TDM-based dose-adjustment strategy may be an effective strategy to enable patients who received a TKI to achieve the target C_min_. The population pharmacokinetics of imatinib in patients with CML in Nigeria (61) showed that treatment with a standard dose of imatinib may not elicit the desired effect in most patients, and that exposure to low concentrations continuously might lead to drug resistance. They suggested the need for a TDM-guided dose- adjustment strategy of imatinib in this population. In summary, those data indicated that dose reduction could be a feasible and safe option for patients with a stable optimal response but who cannot tolerate a standard dose of imatinib (22). If possible, the imatinib concentration in plasma could be monitored to provide an important reference for the dose adjustment of imatinib.

### Dasatinib

Several studies have explored the efficacy and safety of standard-dose dasatinib (100 mg/day) compared with low-dose therapy (<100 mg/day) in clinical trials and real-life settings. A retrospective analysis of the DASISION trial (23) revealed that dose reduction of dasatinib could maintain a superior prevalence of MMR while reducing the risk of dasatinib-related AEs. Of 65 patients with CML (age >65 years) receiving first-line treatment with dasatinib (100 mg/day VS. <100 mg/day),10 patients who required permanent drug withdrawal due to toxicity all received an initial dose of 100 mg/day (24). Iurlo and colleagues (25) retrospectively evaluated 853 CML patients who received dasatinib as first-line and second-line therapy (100 mg/day VS. <100 mg/day). A total of 196 episodes of PE (23.0%) were identified, and 70.4% of PE events were observed in patients who received 100 mg/day.

The CA180-034 study (26) enrolled patients with CML who were resistant and intolerant to imatinib and who were switched to dasatinib. The result of 7-year follow-up indicated that the clinical response at 100 mg/day was similar to that of 70-mg twice daily or 140 mg/day, and was more beneficial in terms of toxicity. Initial half-dose dasatinib therapy (50 mg/day) was suggested to be a safe option for newly diagnosed CP-CML patients. The clinical response and toxicity profile of initial treatment with half-dose dasatinib were more favorable compared with those documented in the DASISION trial (27). The DAVLEC study (28) suggested that low-dose dasatinib (20 mg/day) as the initial dose for older patients with newly diagnosed CP-CML was worthy of consideration.

Dasatinib exposure may be related to the clinical response and toxicity profile. The dose-limiting toxicities (DLTs) and clinical response of dasatinib were analyzed in patients with CP-CML at 17 hospitals in South Korea (62). Those results suggested that the initial dasatinib dose could be reduced to 80 mg/day according to dose adjusted for bodyweight (dose/BW) in South Korean CML patients, especially for those with lower BW. Mizuta and coworkers found that Patients experienced a higher risk of altered treatment with a higher C_min_/D/W (dasatinib concentration adjusted by dose (g), and bodyweight (kg)) (63). Therefore, TDM-guided dose-adjustment strategy may have potential benefits for dasatinib treatment (29). Rousselot and co-workers evaluated whether TDM could reduce the prevalence of dasatinib-induced AEs at 12 months (30). All eligible patients received an initial dose of 100 mg/day of dasatinib, followed by assessment of the C_min_ of dasatinib. Patients were assigned randomly to a dose-reduction strategy (TDM) group and standard-dose strategy (control) group according to C_min_ ≥3 nmol/L. The cumulative prevalence of PE was reduced significantly in the TDM group (15% vs. 4%, 35% vs. 11%, and 39% vs.12% at 1, 2 and 3 years, respectively, p = 0.0094), whereas the prevalence of MMR was similar. A TDM-guided dose-adjustment strategy for dasatinib was feasible and resulted in a significant reduction in the incidence of PE events without impairing the MMR rate upon long-term treatment.

### Nilotinib

The ENESTnd study (64) reported that nilotinib (400 mg, twice daily (bid) VS. 300 mg, bid) had equivalent efficacy, but high-dose therapy led to longer 5-year overall survival compared with imatinib. However, a higher prevalence of cardiovascular events was observed in the high-dose arm. Furthermore, the ENESTswift trial (31) suggested crossover with nilotinib (300 mg, bid) to be efficacious and well tolerated in most patients treated with nilotinib as second-line therapy. In the NILO-RED study (32), patients were recommended to receive dose adjustments to a lower-dose once-daily (qd) regimen after achieving a MMR with standard-dose nilotinib bid schedule (first-line 300 mg, bid; and second-line 400 mg, bid) solely in case of severe toxicity. Switching to a nilotinib qd regimen as maintenance therapy after achievement of MMR on standard-dose schedule is feasible and safe in CP-CML patients regardless of prior treatment history.

## Discontinuation of TKIs therapy

### Imatinib

In recent years, the experience of discontinuation of TKIs therapy in patients with CML has been reported worldwide. A research team in France reported the first key study in 2007 (33). They suggested that a certain proportion of patients with molecular diseases not detected for ≥2 years could discontinue TKIs treatment and maintain molecular remission. One-hundred patients with CP-CML with undetectable molecular disease for ≥2 years were involved in the STIM1 trial (34). Molecular relapse was defined as a significant increase of 1-log reduction or loss of MMR in two consecutive samples. The prevalence of molecular recurrence-free survival was 43% at 6 months and 38% at 60 months, respectively. The cumulative prevalence of molecular recurrence was estimated to be ~60%. Furthermore, 55 patients who suffered molecular relapse achieved a faster DMR after resumption of TKIs treatment, and no patient had disease progression or mutations of the ABL1 kinase domain. Eighty patients with CML who received imatinib treatment were involved in the A-STIM study (35). Molecular relapse was defined as loss of MMR. The TFR prevalence was 64% at 24 months and 61% at 26 months, respectively. In the STIM2 study, 50% of patients continued to have TFR at 24 months (36). Forty patients with CP-CML enrolled in the TWISTER study in Australia (37) received imatinib treatment for >3 years and achieved 4.5-log reduction (MR4.5) for ≥2 years. Molecular relapse was defined as loss of MMR. At 2-year follow-up, the TFR prevalence was 47.1%, and most molecular relapses occurred in the first 4 months after treatment discontinuation. No patient had disease progression or mutations of the ABL1 kinase domain, and imatinib therapy was restarted successfully in all patients who suffered molecular relapse. The Korean Imatinib Discontinuation (KID) study (38) aimed to identify the predictors for safe and successful discontinuation of imatinib therapy, and 90 patients with CML were enrolled. The probability of achieving a sustained MMR at 12 months and 24 months was 62.2% and 58.5%, respectively. The ISAV study in Italy (39) enrolled CML patients with 112 who received imatinib treatment and who had undergone interferon-α treatment previously. If patients maintained MR4.5 for ≥2 years, then imatinib treatment was stopped. In that study, 50.9% of patients lost their MMR. The DOMEST trial (40) was a multicenter phase-II trial conducted in Japan to assess the clinical efficacy and safety of discontinuing imatinib therapy in patients with CML. Patients with sustained MR4.0 for ≥2 years were included. Molecular relapse was defined as the loss of MR4.0, and resumed dasatinib or other TKIs therapy. The prevalence of molecular recurrence-free survival was 69.6%, 68.6%, and 64.3% at 6, 12, and 24 months, respectively.

Lee and collaborators (65) aimed to identify the predictors for successful discontinuation of imatinib therapy, and 48 patients with CP-CML were enrolled. Patients were eligible for therapy cessation after receiving imatinib treatment for ≥3 years, and to maintain undetectable minimal residual disease (MRD) for ≥2 years. That study also included 20 patients who suffered a post-transplant relapse. Molecular relapse was defined as loss of UMRD or MMR. After a median follow-up of 15.8 months, nine patients lost UMRD and MMR in the non-transplant group, whereas all patients in the post-transplantation group maintained UMRD. Previous transplantation, imatinib duration, and UMRD duration were significantly associated with sustained molecular responses. Campiotti and coworkers (66) conducted a systematic review to assess the long-term safety of discontinuation of imatinib therapy in patients with CML. Approximately 50% of patients had TFR, and no death occurred 2 years after discontinuation of imatinib therapy. Those results indicated that discontinuation of imatinib therapy was feasible and safe for patients with CP-CML who had a sustained DMR.

### Dasatinib

STOP-2G (52) was the first multicenter observational study to investigate the feasibility of discontinuation of second-generation TKIs therapy. The discontinuation criteria were patients with ≥3 years for first-line or subsequent lines of dasatinib or nilotinib therapy with sustained MR4.5 for >2 years. Molecular relapse was defined as loss of MMR. Sixty patients were enrolled and the follow-up was 12 months: 43.3% of patients suffered a relapse at a median of 4 months. The TFR prevalence at 1 year and 2 years was 63.3% and 53.6%, respectively. The DADI trial (41) in Japan included 63 patients with CML with a sustained DMR for >1 year. Molecular relapse was considered to be the loss of DMR at any time point. They found that 52.4% of patients experienced a relapse at a median follow-up of 20 months, and all patients regained DMR 6 months after resumption of dasatinib therapy. The first-line DADI trial (42) was a multicenter phase-II trial in 23 Japanese hospitals, and aimed to assess molecular relapse-free survival at 6 months after discontinuation therapy. Fifty-eight patients with CML received dasatinib as first-line treatment and had a sustained DMR for >1 year. Thirty-two patients maintained TFR at 6 months and TFR prevalence at 6 months was 55.2%. The D-STOP trial (43) explored the long-term outcome of 54 patients with CML who stopped dasatinib treatment after achieving a sustained DMR for ≥2 years. At a median follow-up of 16.2 months, 12 patients suffered molecular relapse. The TFR prevalence at 12 months and 36 months was 62.9% and 44.4%, respectively. The DASFREE study (44) enrolled 74 patients with CML who received dasatinib treatment for >2 years and maintained MR4.5 for ≥1 year. At 2-year follow-up, 51% of patients in the first-line-treatment arm and 42% in the second-line-treatment arm continued to have TFR. The prevalence of TFR was 44% for patients who were resistant or intolerant to first-line dasatinib treatment.

A meta-analysis was conducted in patients with CML under a stable DMR to assess the prevalence of TFR and the long-term safety of discontinuation of second-generation TKIs therapy (67). Five single-armed, prospective cohort studies were included, and 517 patients were enrolled. The overall estimated TFR prevalence at a follow-up of 12 months and 24 months was 57% and 53%, respectively. Molecular recurrence occurred mainly in first 12 months after discontinuation therapy. Investigators discovered that 96.5% of patients who resumed TKIs treatment after molecular relapse could achieve MMR rapidly. During 2-year follow-up, four patients died (including two non-CML-related deaths: one died from arterial hemorrhage during the consolidation phase, and the other death was due to heart failure).

### Nilotinib

The phase-II ENESTFreedom trial (45) was the first to evaluate discontinuation of nilotinib therapy. It enrolled 215 patients who received first-line nilotinib treatment and had stable MR4.5 for ≥2 years. All patients continued to receive 1 year of consolidated nilotinib treatment after enrollment, and 190 patients underwent discontinuation of nilotinib treatment. 48.9% of patients maintained TFR at 96-week follow-up. Furthermore, TFR prevalence was closely associated with the Sokal score at the diagnosis (low risk: 61.3%; intermediate risk: 50.0%; high risk: 28.6%). At 5-year follow-up, 81 patients (42.6%) continued to have TFR, and 76 (40.0%) had MR4.5. Patients who suffered a relapse regained MMR (98.9%) and 92.3% had a DMR (68). The STAT2 trial (46) evaluated the efficacy of 2-year consolidated nilotinib (300 mg, bid) therapy for achieving TFR in CML patients with sustained DMR. Molecular relapse was defined as loss of DMR. Fifty-three patients continued to have TFR in the first 12 months among the 78 patients who were eligible to discontinue nilotinib therapy. The TFR prevalence at 3 years was estimated to be 62.8%. Of the 29 patients who suffered a relapse, 25 patients regained DMR after treatment resumption. The ENESTop study evaluated the TFR prevalence in patients with CP-CML treated with TKIs for >3 years and who achieved a sustained DMR after replacing imatinib with nilotinib. The TFR prevalence was 57.9% and 53.2% at 48 weeks and 96 weeks, respectively (47). Treatment-free survival was 52.0% and 46% at 144 weeks (48) and 192 weeks (49). At 5-year follow-up (50), 42.9% (54/126) of patients continued to have TFR. Of the 59 patients who lost the MMR or DMR and were re-introduced to nilotinib treatment, 98.3% regained the MMR, 94.9% regained MR4, and 93.2% regained MR4.5. Overall, AE rates decreased over the 5 years of TFR, and no patients suffered disease progression or CML-related death. The NILSt study (51) enrolled patients with DMR who received nilotinib consolidation therapy for ≤24 months, and who maintained MR4.5 proceeded to discontinuation of nilotinib treatment. Molecular relapse was defined as loss of MR4.5. Eighty-seven patients (58.4%) underwent discontinuation of nilotinib therapy. The TFR prevalence was 60.9% at 1 year and 3 years, respectively. The phase-II study GIMEMA CML 0307 (69) found that 24 (32.9%) patients with a stable DMR discontinued nilotinib treatment at 10-year follow-up, and the TFR prevalence at 2 years was 72.6%. The overall TFR prevalence was estimated to be 24.7%.

LAST (53) was a prospective clinical trial that included 172 patients with CML from 14 academic medical centers in the USA, which aimed to evaluate molecular relapse and patient-reported outcomes after discontinuation of TKIs treatment. Molecular relapse was defined as loss of the MMR. At a median follow-up of 41.6 months, 112 (65.5%) continued to maintained MMR, and 104 (60.8%) achieved TFR. A total of 755 patients were enrolled across Europe in the EURO-SKI trial (54): 94% of patients discontinued imatinib therapy, and 2% and 4% discontinued dasatinib therapy and nilotinib therapy, respectively. Patients received TKIs treatment for ≥3 years and had sustained MR4 for ≥1 year. Relapse-free survival was 61% at 6 months and 50% at 24 months. Disease progression was not observed. The optimal duration of sustained MR4 before treatment discontinuation was 3.1 years calculated by a prognostic model, with 61% probability of retaining MMR. The cutoff for imatinib therapy was 5.8 years, and the molecular relapse-free survival was 63%. Also, 86% of patients regained the MMR after restarting TKIs treatment. The GIMEMA trial enrolled 293 Italian patients with CP-CML who discontinued TKIs therapy (55). 72% patients received imatinib treatment, and the remainder of patients received second-generation TKIs before treatment discontinuation. At 12 months, the TFR prevalence was 68% in the imatinib arm and 73% in the second-generation-TKIs arm. At a median follow-up of 34 months, the overall estimated TFR prevalence was 62%, and disease progression did not occur.

Recently, several retrospective studies have assessed the safety of discontinuation of TKIs therapy outside of clinical trials. One research team (70) enrolled 236 patients with CML from 33 Spanish centers to evaluate the safety of discontinuation of TKIs treatment in a real-life setting. Overall, the TFR prevalence was 64% at 4 years, and no patients suffered disease progression. Most patients who experienced molecular relapse regained the DMR after resuming TKIs therapy for 3–5 months. Iino and coworkers (71) assessed the outcome of 21 patients with CML who discontinued TKIs treatment. The TFR prevalence at 2 years was 66.7%, and no patients experienced disease progression or died. A retrospective study demonstrated that discontinuation of TKIs therapy was safe (especially for patients with a stable DMR with a longer duration of TKI treatment) (72): the prevalence of molecular relapse was 25% in patients with a stable DMR and 85% in those with an unstable DMR. Overall, discontinuation of dasatinib or nilotinib therapy was feasible and safe for patients with a sustained DMR and a longer duration of TKI treatment in clinical trials and real-world settings.

## Dose reduction before therapy discontinuation

The DESTINY study (73) aimed to evaluate the outcome of gradual dose reduction before TKIs discontinuation as well as the safety of TFR for patients with less deep (but stable) remission. In detail, patients from 20 UK hospitals were assigned to a MR4 group and MMR group. TKIs treatment was reduced to half of the standard dose (imatinib = 200 mg/day; dasatinib = 50 mg/day; nilotinib = 200 mg, bid) for 12 months, then discontinued for a further 24 months. Molecular relapse was defined as loss of MMR that necessitated resumption of TKIs treatment at the full dose. The primary endpoint was the proportion of patients who could first experience half-dose therapy for 1 year, and then stop treatment completely for a further 2 years, without losing the MMR. Of the 174 patients, 148, 10, and 16 were treated with imatinib, dasatinib, and nilotinib, respectively. Forty-nine patients were assigned to the MMR group and 125 to the MR4 group. Three patients in the DMR group and nine patients in the MMR group suffered molecular relapse during dose reduction. Eighty-four (67%) patients achieved the primary endpoint and recurrence-free survival was 72% in the DMR group. Sixteen (33%) patients achieved the primary endpoint and recurrence-free survival was 36% in the MMR group. No patients suffered disease progression and two patients died due to unrelated causes. All patients who relapsed regained the MMR within 5 months of resumption of TKIs therapy.

In a retrospective analysis in 2020 (74), 26 patients with CML received a low-dose TKI before discontinuation, and the TFR prevalence at 5 years was 47.5% in the full-dose group and 58.8% in the low-dose group. That study suggested that low-dose TKI regimens before discontinuation of TKI therapy did not impair the chance of achieving TFR in patients with CML. An investigation on the attitude of hematologists practicing in Italy towards a low-dose TKI regimen and its impact on TFR was undertaken (75). Results showed that 64.4% of hematologists believed that TFR should not be affected by low-dose TKIs. Furthermore, this strategy was applied to 194 patients with CML. Except for three patients, all patients reached a DMR with a median treatment duration of 61.0 months at the time of TFR. At a median follow-up of 29.2 months, 138 (71.1%) patients continued to have TFR, and the TFR prevalence was improved significantly after dose reduction due to AEs. However, outside of clinical trials, one-third of Italian hematologists continued to harbor doubts about the safety of TFR after patients received a low dose of TKIs. Interestingly, only 28.9% of patients suffered molecular relapse, which was lower than that reported in the standard dose therapy. That survey suggested that TFR may be an effective and safe option, even in patients who receive treatment with low-dose TKIs. Those findings suggest that low-dose TKIs do not impair the opportunity to achieve TFR. However, more prospective and multicenter clinical trials must be undertaken to explore the efficacy and safety for patients receiving low-dose TKIs before discontinuation of TKI therapy.

## Second attempt to achieve TFR

A second attempt to achieve TFR may be considered for some patients. The details of trials focusing on a second opportunity to achieve TFR are shown in **Table 3**. Ross and collaborators (76) conducted a study on a second discontinuation for 12 patients who regained MR4.5 with restarted treatment after a first molecular relapse. At a median of 8.6 years follow-up, the TFR prevalence was 50%. Patients who relapsed after the first discontinuation of TKIs therapy and who regained a DMR were enrolled in the RE-STIM trial (77). The TFR prevalence after a second attempt at therapy discontinuation was 44.3% at 24 months, 38.5% at 36 months, and 33.2% at 48 months in 70 patients. In the TRAD trial (78), patients who suffered a relapse (loss of MR4 or MMR) after discontinuation of imatinib therapy were resumed on dasatinib therapy (100 mg/day). Patients who regained MR4 that was sustained for >1 year had a second attempt at achieving TFR. The TFR prevalence after a successful attempt at therapy discontinuation was 21.5% at 6 months. In the 2020 A-STIM study (79), 32 (49.2%) patients underwent a second attempt to achieve TFR. The TFR prevalence at the second attempt at treatment discontinuation was 35.8%. Although the TFR prevalence of the second therapy discontinuation was lower than that of the first treatment discontinuation (46.8%), the failure of the first treatment discontinuation did not preclude the success of the second treatment discontinuation. However, patients who lost the MMR rapidly after the first treatment discontinuation had a negligible chance of achieving TFR on a second occasion using TKIs therapy alone.

**Table 3:** Characteristics of secondary TFR trials.

Study	N	TFR rate	Resumed treatment
Ross et.al (76)	40	50% at a median of 8.6 years follow-up	Loss of MMR
RE-STIM (77)	70	44.3% at 24 months, 38.5% at 36 months and 33.2% at 48 months	Loss of MMR
TRAD (78)	25	21.5% at 6 months	Loss of MR4 or MMR
2020 A-STIM (79)	65	46.8% and 35.8% at 1 year and 3 year	Loss of MMR

MMR, major molecular response; TFR, treatment-free remission; UMRD, undetected minimum residual disease; MR, molecular response.

## Switching TKIs

Switching TKIs are required if there are intolerable toxicities, failure to achieve treatment milestones, or a BCR::ABL1 mutation that leads to resistance to specific TKI, (80, 81). The change is mandatory and should be accompanied by BCR::ABL1 KD-mutations tests in cases of failure/resistance. In the absence of BCR::ABL1 KD-mutations, there are no definitive recommendation for any particular TKIs. The criteria for selection of the second-line TKIs are almost entirely patient-related and dependent upon comorbidities, age, and the toxicity of the first TKI. If there is a mutation for a specific TKI, further TKI selection should be select accordingly. In case of warning response, the change is optional, and dependent upon the patients’ long-term treatment goals and personal factors (e.g., age, complications, tolerance and economic situation). In the case of treatment-related complications and intolerance, the decision to switch TKIs is in part subjective, dependent upon the patient, physician, supportive care, and also upon the clinical response levels. The choice of dose of converted TKIs must take into account the clinical response and tolerance of the patient, as well as the standard- or reduced-dose regimens.

## Pediatrics CML

In addition to imatinib, dasatinib and nilotinib were approved recently for pediatric CML treatment, which has expanded the therapeutic options. Moreover, allogeneic stem cell transplantation suggest to be third-line treatment for most pediatric cases (82). However, children are actively growing during TKIs treatment, so they develop unique AEs, such as growth disturbance (83). Currently, there are lacking of sufficient data on efficacy and safety to pediatric patients, TKI selection mostly to be reliant on the clinical effects observed in adults.

Some research teams based in European groups recommend a lower starting dose of imatinib in children with CP-CML (260–300 mg/m^2^/day) (84). However, Children’s Oncology Group CML Working Group suggested a higher dose of imatinib was also well tolerated (85), and the initial recommended dose is 340 mg/m^2^/day (maximum to 600 mg). The initial dose of dasatinib is 400-100 mg/m^2^ qd (maximum to 100 mg) in children with CP-CML (86), and 230 mg/m^2^/dose bid for nilotinib (maximum single dose of 400 mg) (87). The dose should be recalculated every 3 months based on changes in body-weight or more frequently if required, and could be adjust on the basis of clinical response and tolerability, but the maximum dose should not be exceeded.

Limited evidence regarding discontinuation of TKIs therapy is available for pediatric CML, mostly in small studies and case series. The Japanese Pediatric Leukemia/Lymphoma Study Group (88) reported the first prospective pediatric discontinuation of TKIs trial in 22 patients with CP-CML who had been taking TKIs for >3 years and had sustained MMR (MR4.0) for >2 years. TFR at 12 months was 50%. Of seven pediatric patients who discontinued imatinib, two patients achieved TFR (85). The STOP IMAPED study enrolled 14 pediatric patients who were treated with imatinib for ≥3 years and sustained DMR for ≥2 years to discontinued imatinib, the TFR rate at 6 months was 28.6% (89). Millot and colleagues (90) reported a TFR rate of 56% at 36 months after discontinuation of imatinib in 18 pediatric patients with sustained DMR for ≥23.9 months. Shima and coworkers (91) evaluated the feasibility of discontinuation of TKIs in pediatric CML patients. Twenty-two patients were eligible to discontinue TKIs if they treated with TKIs for ≥3 years, and sustained MR4.0 for ≥2 years. Their results showed the TFR rate to be 50.0% at 12 months, and that all patients regained MR4.0 after resumption of TKIs therapy. Therefore, discontinuation of TKIs therapy in pediatric CML is not recommended outside of clinical trials, and more prospective studies in pediatric CML are needed.

## Conclusions

Recently, increasing numbers of patients with CML have begun to pursue a dose optimization strategy, which included dose reduction and discontinuation of TKIs therapy. In the real-life settings, we will comprehensively consider the dose optimization strategy base on the treatment goal, clinical response, tolerance, and economic situation of patients. A dose-reduction regimen could allow for broader clinical use of TKIs (even in patients with comorbidities). For example, if the elderly patient with multiple comorbidities or is previously intolerant to other TKIs, we may suggest a half-dose of dasatinib treatment (50 mg/day). If conditions permit, the dose can also be adjusted according to blood concentration monitoring. For patients with sustained optimal clinical response (MMR or DMR), reducing TKIs dose can reduce AE and improve treatment compliance. The proposal of TFR as a possible final treatment endpoint should be discussed with patients (especially younger patients) at the diagnosis to achieve a DMR rapidly and improve long-term compliance. For patients with stable DMR and long duration of TKIs treatment, it is feasible and safe to stop TKI treatment. Patients who discontinued TKIs should follow the discontinuation standards recommended in ELN or NCCN guidelines. For patients with stable DMR who want to stop TKIs treatment but are afraid of relapse, we recommend to reduce TKIs dose before discontinuation of TKIs therapy. For patients who cannot achieve TFR, the TKIs dose must be reduced without affecting the clinical response. Importantly, patients who underwent dose optimization should be advised for more intensive molecular monitoring, especially during the first 6 months. Once the patients lose the optimal response, physicians should take measures immediately, such as resuming to standard-dose therapy, reintroducing TKIs treatment, or switching to other TKIs, etc. However, evidence for dose optimization in pediatrics CML is limited. Hence, evidence from novel, prospective clinical trials and real-life clinical practice are required to explore dose-optimization strategies, which may provide more promising options for CML treatment.

## Author contributions

FC: Writing (original draft) and data curation. QL: Validation, data curation, and writing (original draft). ZC: Data curation. MH: Revision of the original draft. WL: Conceptualization, visualization, and data curation. YZ: Project administration and funding acquisition. All authors contributed to the article and approved the submitted version.
